# Antihypertensive potential of *Brassica rapa* leaves: An *in vitro* and *in silico* approach

**DOI:** 10.3389/fphar.2022.996755

**Published:** 2022-09-30

**Authors:** Rohma Abid, Muhammad Islam, Hamid Saeed, Abrar Ahmad, Fariha Imtiaz, Anam Yasmeen, Hassaan Anwer Rathore

**Affiliations:** ^1^ Section of Pharmaceutical Chemistry, University College of Pharmacy, Allama Iqbal Campus, University of the Punjab, Lahore, Pakistan; ^2^ Section of Pharmaceutics, University College of Pharmacy, Allama Iqbal Campus, University of the Punjab, Lahore, Pakistan; ^3^ Section of Pharmacognosy, University College of Pharmacy, Allama Iqbal Campus, University of the Punjab, Lahore, Pakistan; ^4^ Department of Pharmaceutical Sciences, College of Pharmacy, QU Health, Qatar University, Doha, Qatar

**Keywords:** *Brassica rapa*, food, antihypertensive action, ACE inhibitor and molecular docking, in silico

## Abstract

**Aim:** Plants contain many essential constituents and their optimization can result in the discovery of new medicines. One such plant is *Brassica rapa* that is commonly used as a vegetable to fulfill daily food requirements worldwide. This study intends to screen the phytochemicals, antihypertensive potential, GC-MS, and *in silico* analysis of the leaves of *Brassica rapa*.

**Methods:** Powdered leaves were subjected to proximate analysis followed by estimation of primary metabolites. Extracts were obtained by hot and cold extraction and investigated for secondary metabolites. All crude extracts were screened for their antihypertensive potential using an angiotensin-converting enzyme (ACE) inhibition assay. GC-MS analysis was carried out to standardize the extract, and an antihypertensive metabolite was confirmed using an *in silico* approach.

**Results:** Physicochemical evaluation resulted in moisture content (9.10% ± 0.1), total ash value (18.10% ± 0.6), and extractive values (water 9.46% ± 0.5 and alcohol soluble 4.99% ± 0.1), while phytochemical investigation revealed primary metabolites (total proteins 11.90 mg/g ± 0.9; total fats 3.48 mg/g ± 0.5; and total carbohydrates 57.45 mg/g ± 1.2). Methanol extract showed the highest number of secondary metabolites including polyphenols 93.63 mg/g ± 0.6; flavonoids 259.13 mg/g ± 0.6; and polysaccharides 56.63 mg/g ± 1.4, while water extract (70 mg/g ± 2) was rich in glycosaponins. Methanol extract showed the highest antihypertensive potential by inhibiting ACE (79.39%) amongst all extracts, compared to the standard drug captopril, which inhibited 85.81%. Standardization of methanol extract *via* GC-MS analysis revealed potent phytoconstituents, and a molecular docking study confirmed that oleic acid is the main antihypertensive metabolite.

**Conclusion:** We conclude that leaves of *Brassica rapa* can successfully lower hypertension by inhibiting ACE, however; *in vivo* investigations are required to confirm this antihypertensive activity.

## 1 Introduction

Despite great development in the field of medicine, the disease burden is high. Plants contain many essential constituents, optimization of which can result in the discovery of new medicines. Drugs obtained from natural sources like plants are safer and economically viable. The primitive and current research evidence suggests that plants are a major source and popular medicine for maintaining health in people of the Asian subcontinent (Sheng-Ji, 2001). Drugs derived from natural origin mainly plants as food have garnered attention as potential therapeutic entities (Shoeb, 2006).

Plants included in the Brassicaceae or Cruciferae family are generally common vegetables that fulfill the daily food requirements throughout the world. The roots and green leaves of such a plant, *Brassica rapa,* are commonly called turnip and have nutritional and medicinal values (Paul et al., 2019). According to the United States Department of Agriculture’s Food Data Central database, one cup of raw turnip cubes contains around 36.4 calories, 1.17 g of protein, 0.13 g of fat, 8.36 g of carbohydrates including 4.66 g of sugar, 2.34 g of fiber, 39 mg of calcium, 0.39 mg of iron, 14.3 mg of magnesium, 35.1 mg of phosphorus, 0.13 µg of vitamin K, 87.1 mg of sodium, 0.351 mg of zinc, 27.3 mg of vitamin C, and 19.5 µg of folate. *Brassica rapa* has a variety of volatile substances such as aldehydes, ketones, alcohols, and esters. It also contains flavonoids and derivatives of hydroxycinnamic acid. Turnips are also popular as fodder for animals (Taveira et al., 2009). In Perso-Arabic tradition, *Brassica rapa* was employed as a medicine for inflammation of the gall bladder, gall stones, constipation, hepatic diseases, and gastritis. The roots of turnip possess antibacterial properties and were used in the treatment of common cold (Beltagy, 2014). For a long time, the powder of seeds of *Brassica rapa* is well-known for its anticancer properties, especially in breast cancer, and the product obtained from roots has shown benefits in skin cancer. Turnip has also been a common diet and herbal remedy in Tibet, where high altitude leads to oxygen deficiency and exhaustion (Ahmadvand and Sariri, 2008).

Norlander et al. (2018) explained hypertension with reference to the current recommendation as an increase in systolic blood pressure of more than 130 mm Hg. The prevalence of primary hypertension is almost 95% and is associated with cardiac diseases, diabetes, and hyperlipidemia (Messerli et al., 2007). About 24% of the total population or 43 million individuals in the United States of America are hypertensive (Carretero and Oparil, 2000), and nearly 25% of the residents in developed countries are suffering from the problem of raised blood pressure (Lifton et al., 2001). It is estimated that by 2025, the worldwide cases of hypertension may rise to 500 million (Norlander et al., 2018).

With a population of more than 150 million, Pakistan is dealing with the widespread issue of elevated blood pressure. In consonance with a survey study carried out in Pakistan, three-quarter of the natives is suffering from hypertension without being aware of it (Ahmad and Jafar, 2005). Recent analysis has indicated the prevalence of hypertension in Pakistan to be 20.7%, whereas high blood pressure is still manageable in underdeveloped countries with proper management of the disease (Mittal and Singh, 2010). Lifestyle choices and other major factors such as increased consumption of salt and liquor, sedentary lifestyle, being overweight, etc., are important contributors to the high prevalence of hypertension. Further causes responsible for hypertension include anxiety, traumas, and miscellaneous ecological aspects (Whelton, 1994). Diuretics, calcium channel blockers, RAAS inhibitors, and beta-blockers are mostly the drugs of choice prescribed as a single pill or in combination therapy to the population facing high blood pressure. However, their continuous use leads to many side effects, including erectile dysfunction, nervousness, muscle weakness, and frequent headaches (Williams, 2009).

Hypertension can lead to heart problems such as kidney failure, coronary heart disease, and peripheral arterial disease. Pharmacological approaches for alleviating the symptoms of hypertension tend to decline the incidence of cardiac diseases including myocardial infarction, stroke, etc. However, for the elimination and cure of hypertension, non-pharmacological therapy could be the most effective approach (Burt et al., 1995). Comini et al. (2007) stated that ACE inhibitors exert their effect by over-expression of an endothelial enzyme, nitric oxide synthase (eNOS), which is involved in the regulation of blood pressure by producing nitric oxide, a potent vasodilator.

Literature shows several studies where turnip has been investigated for many pharmacological activities, but the antihypertensive activity of the leaves of *Brassica rapa* has not been investigated. Thus, the purpose of the current study is to analyze the leaf extract of *Brassica rapa* for antihypertensive activity by applying an angiotensin-converting enzyme (ACE) inhibition assay, followed by *in silico* analysis.

## 2 Materials and methods

### 2.1 Collection of plant leaves

Approximately 15 kg leaves were collected in January 2021 from the fields of Gujranwala district of northern Punjab. The leaves were washed, and dust was removed. They were dried in sunlight for 4–5 days. After drying, the leaves were crushed to a fine powder. The powdered form weighed almost 2,500 g and was stored in an air-tight jar. The identification and authentication of leaves were carried out by Prof. Dr. Zaheer-ud-din Khan from the Botany Department of Government College University, Lahore (GCU). Voucher number GC. Herb.Bot.3721 was allocated by the GCU Lahore herbarium to the plant.

### 2.2 Solvent and chemicals

N-hexane (Merck, United States), chloroform (May and Baker, UK), methanol (Merck, Germany), ethanol (Merck, Germany), deionized water, hydrochloric acid (BDH, England), sulphuric acid (BDH, England), nitric acid (BDH, England), sodium carbonate, copper sulfate, potassium acetate, aluminium nitrite, acetone, sodium hydroxide, sodium chloride (E. Merck A. G Darmstadt, Germany), quercetin (Sigma, United States), bovine serum albumin (Sigma, United States), gallic acid (Sigma, United States), anhydrous glucose (Merck, Germany), Triton X (Sigma, United States), Folin–Ciocalteau’s phenol reagent (Unichem Chemicals, India), anthrone reagent (Sigma, United States), monosodium phosphate (BDH, England), disodium phosphate (Riedel-de Haen, Germany), 3, 5-dinitrosalicylic acid (BDH, England), potassium sodium tartrate (BDH, England), and distilled water were used in experiments.

### 2.3 Preparation of plant extract

The hot and cold extraction method was used for collecting the extract from the leaves of *Brassica rapa.* Hot extraction was carried out using the Soxhlet apparatus. The extraction was carried out by using the solvents in increasing order of polarity, that is, n-hexane, chloroform, and methanol. Cold extraction was simply carried out using two polar solvents: alcohol and distilled water (Yadav and Agarwala, 2011).

### 2.4 Physicochemical analysis

Physicochemical analysis was performed on the leaves of *Brassica rapa* with reference to the protocols of USP (2005).

#### 2.4.1 Moisture content

Two grams of powdered leaves were weighed and placed in the crucible. The crucible was then placed in an oven at 105 °C for 30 min. After 30 min, the crucible was taken out and weighed. The loss in weight was recorded. The method was repeated until the weight of the powder became constant. Calculation of moisture content was carried out by using the following formula (Eq. A.1, A.2):

Dry matter (%)=Initial weight−final weight/weight of dried sample×100, (A.1)

Moisture content (%)=100−Dry matter. (A.2)

#### 2.4.2 Ash values

##### 2.4.2.1 Total ash

Two grams of powdered sample were taken in a pre-weighed China dish. China dish was incinerated by placing it in a muffle furnace at 675 ± 25°C until the sample became carbon-free. It was taken out of the furnace and allowed to cool in a desiccator at room temperature. Weight was noted, and the following formula (Eq. B.1) was used to calculate the total ash content:

Total Ash (%)=weight of Ash/total weight of powder×100. (B.1)

##### 2.4.2.2 Acid insoluble ash

Two grams of sample were measured and placed in a China dish in a muffle furnace at 675°C until it became carbon-free. It was then cooled in desiccators and weighed. The resultant ash was boiled for 5 min in 25 ml of 3N HCL and allowed to cool at room temperature. The mixture was filtered using ashless filter paper. The residue obtained was washed with hot double distilled water and again placed in the furnace. The sample was cooled and weighed when it became carbon-free. The percentage of acid insoluble ash was determined by the following formula (Eq. B.2):

$$Acid\;Insoluble\;Ash\;\left(\%\right)=weight\;of\;acid\;insoluble\;\frac{ash}{total}\;ash\;weight\times 100.$$ (B.2)

##### 2.4.2.3 Water insoluble ash

The ash obtained in the total ash test was boiled for 5 min with 25 ml of double distilled water and passed through ashless filter paper. The residue left on the filter paper was placed in a China dish which was allowed to incinerate in a muffle furnace at 450°C for 15 min. The weight of China dish was then measured, and an assessment of water-insoluble ash was carried out by the following formula (Eq. B.3):

Water Insoluble Ash (%)=weight of water inssoluble ash/total ash weight×100. (B.3)

##### 2.4.2.4 Sulfated ash

Two grams of sample were added to a washed, dried, and pre-weighed china dish. Then, 1 ml of concentrated sulphuric acid was added. Also, it was heated on a low flame until the fumes disappeared. This process was repeated twice, and then the China dish was placed in the muffle furnace at a temperature of 600 ± 25°C for 30 min. The China dish was cooled and weighed. The percentage of sulfated ash was measured using the following equation (Eq. B.4):

Sulfated Ash (%)=weight of sulfated ash/weight of powder ash×100. (B.4)

#### 2.4.3 Extractive values

##### 2.4.3.1 Water soluble extractive value

Five grams of powder were accurately weighed and added to a conical flask containing 100 ml of double distilled water. The flask with a magnetic stirrer was placed on the hotplate to allow shaking for 24 h. The solution was filtered after 24 h. China dish was weighed and 25 ml of the filtrate was added and placed in an oven at 105°C for evaporation. After evaporation, the China dish was weighed again. Extractive value relative to dried powder was evaluated. The percentage was calculated by the following equation (Eq. C.1):

Water soluble extractive value (%)=weight of dried extract/weight of powder sample×100. (C.1)

##### 2.4.3.2 Alcohol soluble extractive value

Five grams of powder from the leaves of *Brassica rapa* was mixed with 100 ml of ethanol in a conical flask and was continuously shaken on a hotplate. After 24 h, it was removed from the hot plate and filtered. Twenty-five mL of filtrate in the China dish was then placed in the oven at 105°C. The weight of dried material was recorded with reference to the weight of the air-dried sample. The percentage of alcohol soluble extractive value was measured by the formula given below (Eq. C.2):

Acid soluble extractive value (%)=weight of dried extract/weight of powder sample×100. (C.2)

### 2.5 Estimation of primary metabolites

#### 2.5.1 Total protein content

Estimation of total protein content was completed by following the protocol of Lowry et al. (1951) with slight modifications. In a Falcon tube, 1 g of powdered material, 10 ml of distilled water, and five drops of Triton X were added and shaken randomly. The solution in the Falcon tube was centrifuged at 2,700 rpm for 10 min. After centrifugation, the supernatant layer was formed, from which 100 µl was taken and double distilled water was added to make the volume up to 1 ml. To this mixture, 3 ml of reagent C and 0.2 ml of Folin–Ciocalteu reagent were added. Reagent C was prepared by combining 50 ml of reagent A and 1 ml of reagent B. Reagent A consisted of 2% sodium carbonate and 0.1 N sodium hydroxide while reagent B comprised 0.5% copper sulfate in 1% potassium sodium tartrate. The sample was incubated at room temperature for 30 min. Absorbance against a blank solution was taken at 600 nm. The blank solution was prepared in the same way as the sample solution except for the powdered sample. Bovine serum albumin (BSA) was used as a standard for protein content estimation. The absorbance of different dilutions ranging from 20–120 μg/ml was measured and plotted against the standard curve through linear regression.

#### 2.5.2 Total lipid content

For hot extraction with n-hexane, 50 g of powdered material was packed in a thimble, and maceration was carried out in the solvent. The temperature of 40–60°C was maintained throughout the process of extraction. After completion of extraction, filtration was carried out, and a rotary evaporator was used to dry the filtrate. It was then transferred to a pre-weighed glass vial. The vial was then placed in an oven at 40°C for further drying. Evaluation of total lipid content was carried out after it was completely dried and weighed and expressed in mg/g of the total sample taken (Besbes et al., 2004).

#### 2.5.3 Total carbohydrates

The formula discussed by Al-Hooti et al., 1997 for total carbohydrate determination was used by taking the difference between total lipid and total protein content from 100 (Eq. D.1).

Total carbohydrates (%)=100−(Total moisture+Total ash+Total fats+Total proteins). (D.1)

### 2.6 Estimation of secondary metabolites

#### 2.6.1 Determination of total polyphenols

Evaluation of phenolic content of all the five extracts of *Brassica rapa* leaves was accomplished in accordance with the experiments by Slinkard and Singleton (1977). The standard used for plotting the calibration curve in this experiment was gallic acid. Standard and stock solutions were prepared using a 1 mg/ml concentration of methanol. In individual Falcon tubes, 200 µL of sample and standard were taken. Then, 200 µL of Folin–Ciocalteu (FC) reagent was added. Two mL of 15% sodium carbonate was added after 4 min, and the final volume was achieved with 3 ml methanol. A blank solution was made which contained the same reagents except for the sample. Then, 10, 20, 40, 60, 80, 100, and 120 μg/ml dilutions of the sample were prepared. All the samples, blank and standard were incubated at room temperature for 2 h and absorbance was found using a UV–visible spectrophotometer at 760 nm. Total phenolic content was measured through a linear regression equation and expressed as mg/g.

#### 2.6.2 Determination of total flavonoids

The number of flavonoids in *Brassica rapa* leaves was measured by using a method proposed by Chang et al. (2002). For the preparation of a standard solution of quercetin, methanol was used. Stock solutions of all the five extracts of samples were prepared using methanol at a concentration of 1 mg/ml. Different dilutions of the sample were made. The working solution was prepared by mixing 200 µl solution from the stock solution and standard with methanol, and the volume was made up to 1 ml. After that 100 µl of 10% (w/v) aluminum nitrate, 100 µl 1 M potassium acetate, and 4.6 ml of double distilled water were added. The blank solution had all reagents except the sample. For measuring the absorbance, all test tubes were incubated at 25°C, and absorbance was recorded using a UV spectrophotometer at a wavelength of 415 nm. Using quercetin as a reference standard, total flavonoids were then calculated.

#### 2.6.3 Total polysaccharides

The procedure given by Hussain et al. (2008) was used for total polysaccharide content. A quantity of 200 mg taken from each of the five extracts was mixed in 80% of 7 ml of warm ethanol. The solution was run for 2 min on a vortex mixer and then centrifuged at 2,700 rpm for about 10 min. Anthrone reagent (45 mg anthrone in 100 ml chilled 85% sulphuric acid) was added drop-wise to the residue, and the same process was repeated until the solution displayed no color upon the addition of anthrone reagent. The residue was then dried, and 10 ml digestion mixture (5 ml of 25% HCl and 5 ml of double distilled water) was added. The Falcon tubes were put in an ice bath for 20 min until the temperature reached 0°C. This was followed by centrifugation for 10 min, and the supernatant was obtained. For the collection of supernatants, the whole process was repeated. The final volume was made up using distilled water; 4 ml of anthrone was added to the mixture, which was boiled in a water bath for 8 min and then chilled instantly. The blank solution did not contain the sample but contained water, ethanol, anthrone, and digestion mixture. Absorbance was taken at 630 nm by using glucose as standard. Different dilutions ranging between 10 and 120 µL were prepared for all the extracts, absorbance was recorded, and a standard curve was plotted. The linear regression equation was used to find out the values, and the results were multiplied by a factor of 0.9.

#### 2.6.4 Total glycosaponins

Siddiqui et al. (2009) stated that 1 g of extract was taken in a 100-ml round-bottom flask, and 50 ml of methanol was added. The solution was allowed to heat on a reflux condenser for 30 min. It was repeated two times and then filtered. The filtrate was passed through the rotary evaporator until much of it evaporated, leaving behind 10 ml of filtrate in the flask; 50 ml acetone was taken in a beaker, and 10 ml of the filtrate was added drop-wise. Consequently, saponins were precipitated out. Precipitates were dried in an oven at 100°C until the weight became constant. The same method was used for the rest of the extracts. The formula used for the estimation of glycosaponins is stated below (Eq. E.1):

Glycosaponins (mg/g)=weight of precipitate/weight of sample×100. (E.1)

#### 2.6.5 Fourier transform infrared (FTIR) profiling

Functional group analysis in leaves of *Brassica rapa* was completed using a Fourier transform infrared spectroscopic technique. All the extracts of leaves of *Brassica rapa* were placed on a crystal of the FTIR spectrophotometer, and spectra were recorded.

### 2.7 *In vitro* antihypertensive activity

#### 2.7.1. Preparation of reagents

• Reagent A: 300 mM sodium chloride with pH 8.3 in 100 mM sodium borate buffer
• Reagent B: Buffer substrate solution (using 5 mM hippuryl-L-histidyl–L-leucine (HHL) and reagent A)
• Reagent C: Angiotensin-converting enzyme (ACE) solution (0.1 unit/ml).

#### 2.7.2 Preparation of sample and standard stock solutions

Sample solutions of n-hexane, chloroform, methanol, ethanol, and water extract were made by taking 1 mg of each extract in 1 ml of sodium borate buffer pH 8.3. The reference standard solution was made using 1 mg of captopril in 1 ml of sodium borate buffer pH 8.3.

#### 2.7.3 Angiotensin-converting enzyme (ACE) inhibition assay

The ACE inhibition assay proposed by Cushman and Cheung (1971) was performed with slight modifications. The sample for each extract was prepared by mixing 100 µl buffer substrate solution (reagent B), 40 µl extract solution, and 20 µl ACE solution (reagent C). For control, 100 µl of buffer substrate solution (reagent B), 40 µl of deionized water, and 20 µl of ACE solution (reagent C) were mixed together. Preparation of blank solution requires 100 µl of buffer substrate solution (reagent B) and 60 µl of deionized water; 100 µl of buffer substrate solution (reagent B), 40 µl of captopril solution, and 20 µl of ACE solution (reagent C) were taken to make the standard solution. All the test tubes were incubated for 30 min, and the temperature was maintained at 37°C. To end the reaction, 250 µl of 1M HCl was added. The formation of hippuric acid occurs as a result of the reaction between angiotensin-converting enzyme (ACE) and hippuryl-L-histidyl -L-leucine (HHL). For extraction of hippuric acid, 1 ml of ethyl acetate was used and subjected to vigorous stirring in a vortex mixer for 15 s. Centrifugation of all samples was completed in 10 min. One mL of the organic layer was shifted to a test tube and heated at 100°C for 30 min until it evaporated. The residual matter was again dissolved in 1 ml of deionized water. Using a spectrophotometer, the absorbance of the sample, blank, control, and standard was measured at 228 nm. The percentage inhibition was calculated by using the following equation (Eq. F.1):

Percentage inhibition (%)=test control−test solution/test control−blank solution×100. (F.1)

### 2.8 GC-MS analysis of methanol extract of leaves of *Brassica rapa*

GC-MS (GC system 7890A, Agilent Technologies, United States, and MS 5975C, Agilent Technologies, United States) was used for the analysis of methanolic extracts of powdered leaves of *Brassica rapa.* The capillary tube was 30 m in length and 0.25 nm in diameter with a film coating of 0.25 μm. A carrier gas, helium, with a flow rate of 0.25 ml/min was used. The extract was dissolved in methanol for preparing the sample. A sample volume of 1 μl was taken, and column pressure was set to 0.77 psi. Initially, for 5 minutes, the temperature of the oven was maintained at 100°C, which was then raised to 200°C at the rate of 10°C/min and retained for 10 min. In the end, at the rate of 25°C/min, the temperature was elevated to 325°C for 30 min and was kept constant for 10 min. Various peaks were obtained for different compounds present in the methanolic extract of *Brassica rapa* leaves. The constituents present in the extracts were interpreted by comparing the peaks to the database of the National Institute of Standards and Technology NIST20, having a minimum quality factor of 70 (Priya and Subhashini, 2016).

### 2.9 *In silico* molecular docking studies

Molecular modeling was performed on the selected compound using the Schrödinger Maestro Suite.

#### 2.9.1 Preparation of protein

Three-dimensional conformation of the protein, that is, eNOS (PDB: 1D0C), was achieved with the help of the Protein Preparation Wizard in Maestro. All the water molecules and ligands were removed from the protein structure.

#### 2.9.2 Preparation of ligand

Oleic acid, obtained in GC-MS analysis, was selected as a ligand. For molecular modeling, a minimized structure was preferred.

#### 2.9.3 Molecular docking analysis

Using Glide software available in Maestro, molecular docking of the selected ligand, that is, oleic acid, was carried out. Initially, for locating the binding site with the highest affinity, blind docking was conducted, and finally molecular docking was conducted using Glide 6.2 Extra Precision.

#### 2.9.4 Molecular dynamic simulation

For an improved understanding of compounds, molecular modeling was performed for 50 ns. In the first step, using the antechamber module of Amber18, libraries, specifications for the receptor, and the ligand were generated. Charge neutralization, hydrogen atom minimization, and optimization of energy were achieved. For limitation of the hydrogen bond system, the temperature was raised to 300 K *via* canonical (NVT) ensemble. Keeping pressure constant through NPT equilibration was accomplished for 100 ps. Finally, the system was examined for stability using systematic simulations (Wahedi et al., 2021).

## 3 Results

### 3.1 Extraction and proximate analysis

Results of proximate analysis of *Brassica rapa* are mentioned in Table 1. Moisture content found in the leaves of *Brassica rapa* was 9.1%. Powdered roots had a percentage moisture content of 6.48 ± 0.45%, and the total ash noted in sample leaves of *Brassica rapa* was 18%. The acid insoluble ash was found to be 5.7%, water-soluble 5.20% and sulfated was 21.60%.

**TABLE 1:** Proximate analysis of extracts of *Brassica rapa* leaf powder.

Sr no.	Physicochemical parameters	Percentage content ±SD (% w/w)
1	Moisture content	9.1 ± 0.1
2	Total ash	18.10 ± 0.6
3	Acid insoluble ash	5.70 ± 0.2
4	Water soluble ash	5.20 ± 0.2
5	Sulfated ash	21.60 ± 0.6
6	Alcohol soluble extractive	4.99 ± 0.1
7	Water soluble extractive	1.1 0.5

### 3.2 Determination of phytochemicals

#### 3.2.1 Determination of primary metabolites

The primary metabolite content in all extracts of *Brassica rapa* leaves was resolute and is listed in Table 2. For determination of protein content, linear regression equation was employed Y = 0.0101x + 0.0296, R 2 = 0.9452, and calibration curve was plotted for (10, 20, 40, 60, 80, and 100 μg/ml) dilutions of bovine serum albumin (BSA).

**TABLE 2:** Primary metabolites (mg/g) of powdered leaves of *Brassica rapa*.

Sr no.	Primary metabolites	mg/g ± SD
1	Total proteins	11.90 ± 0.9
2	Total lipids	3.48 ± 0.5
3	Total carbohydrates	57.45 ± 1.2

#### 3.2.2 Determination of secondary metabolites

The secondary metabolite content in all extracts of *Brassica rapa* leaves was resolute and is listed in Table 3. Analysis of all secondary metabolites through linear regression equations was performed using different standards for different metabolites. By linear regression equation, total protein content was calculated as y = 0.0101x + 0.0296, *R* ^2^ = 0.9452. Bovine serum albumin was used as standard, and a calibration curve was plotted. The highest protein content was exhibited by methanol extract of leaves, followed by water, ethanol, chloroform, and n-hexane. For evaluation of total polyphenols, regression equation used was y = 0.0037x + 0.0178, *R* ^2^ = 0.9953. Gallic acid was used as a standard for methanol extract with maximum phenolic content. Using standard curve of quercetin, the total flavonoid count was y = 0.0024x + 0.0147. The highest value for flavonoids was observed in methanol.

**TABLE 3:** Secondary metabolites (mg/g) of extracts of powdered leaves of *Brassica rapa*.

Extracts	Total proteins mg/g	Total polyphenols mg/g	Total flavonoids mg/g	Total polysaccharides mg/g	Total glycosaponins mg/g
n-hexane	9.78 ± 1.2	15.82 ± 0.7	28.54 ± 0.5	21.53 ± 0.4	Negative
Chloroform	13.68 ± 1.2	74.65 ± 0.7	151.93 ± 1.0	26.30 ± 0.5	Negative
Methanol	56.29 ± 1.1	93.63 ± 0.6	259.13 ± 0.6	56.63 ± 1.4	60.13 ± 1.6
Ethanol	35.53 ± 0.6	37.38 ± 1.2	172.68 ± 0.5	43.16 ± 1.0	53.26 ± 0.6
Water	37.18 ± 0.9	46.66 ± 0.5	105.08 ± 0.4	19.02 ± 0.4	70 ± 2

Total polysaccharide estimation was carried out using a linear regression equation y = 0.0028 + 0.022, *R* ^2^ = 0.9957, where glucose was used as a standard. The results showed that the extract of methanol has polysaccharides in a large amount. According to our study, water extract has the highest content of saponins.

#### 3.2.3 FTIR scanning

**FIGURE 1:**
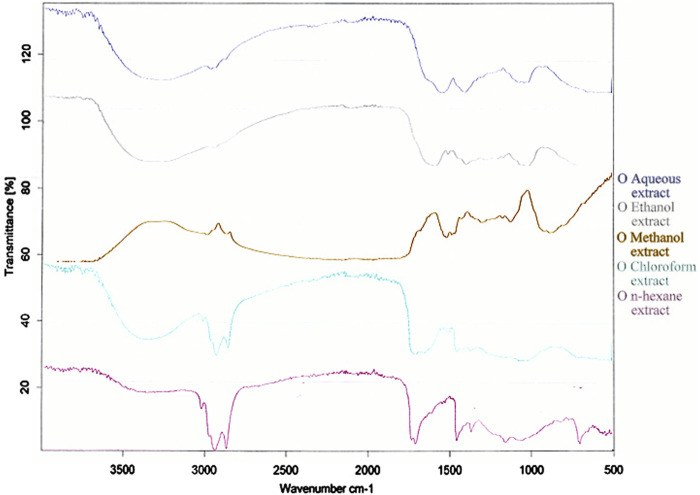
FTIR spectrum of extracts of leaves of *Brassica rapa*.

FTIR scans of five extracts of *Brassica rapa* are presented in Figure 1. There was a broad range of peaks at the 3,500–3,200 cm^−1^ region in n-hexane and ethanol extracts which indicated O-H stretching. Methanol and water extracts showed small but distinct peaks, whereas all three extracts of n-hexane, chloroform, and ethanol showed distinct and sharp peaks in the 3,000–2,500 cm^−1^ range for C-H stretching bonds. All extracts have shown a series of peaks in the region of 2,140–1990 cm^−1^, which shows N=C=S stretching.

#### 3.3.4 Estimation of ACE inhibition activity of extract of leaves of *Brassica rapa*

**FIGURE 2:**
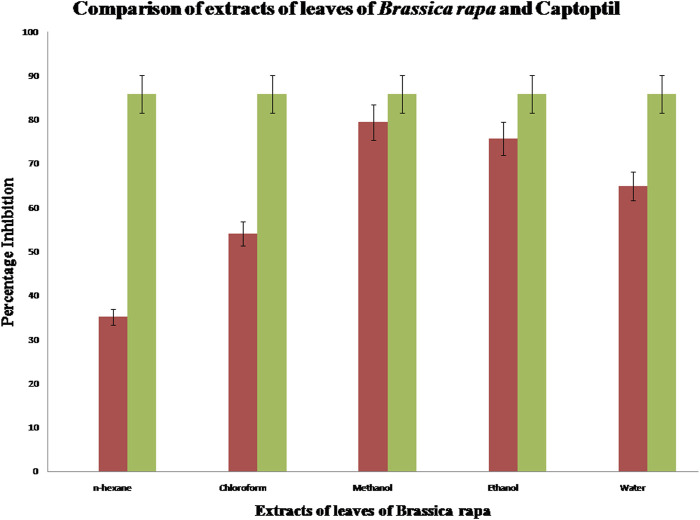
Comparison of ACE inhibition activity of extracts of leaves of *Brassica rapa* with standard captopril.

Angiotensin-converting enzyme (ACE) inhibition activity of a concentration (1 mg/ml) of various leaf extracts of *Brassica rapa* was analyzed. The percentage inhibition of each solution is shown in Figure 2. It is evident from the results that methanolic extract of leaf powder of *Brassica rapa* shows the highest percentage inhibition of angiotensin-converting enzyme. Hence, the methanol extract has an activity against hypertension that is comparable to the ACE inhibition activity achieved with the standard drug captopril, while n-hexane has a 35% inhibitory effect that is least comparable to the reference standard. Further *in vivo* studies using other models can be conducted on the leaf extract of *Brassica rapa* to investigate the antihypertensive potential of the plant.

#### 3.3.5 Estimation of GC-MS analysis of methanol extract of leaves of *Brassica rapa*

**FIGURE 3:**
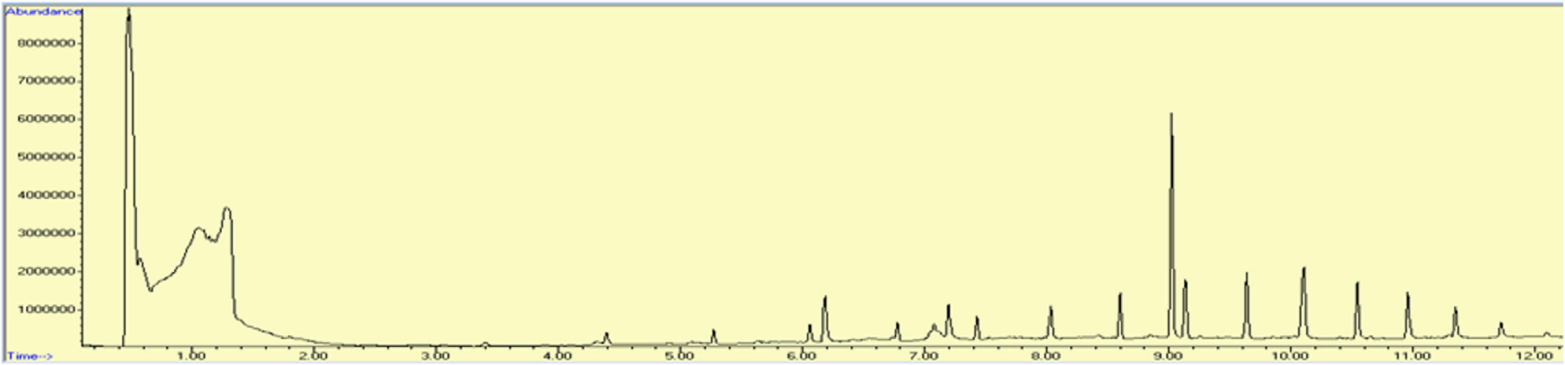
Chromatogram obtained by GC-MS analysis representing retention time, percentage area, molecular formula, and molecular weight of compound present in methanolic extracts of leaves of *Brassica rapa*.

The chromatogram shown in Figure 3, names of the compounds obtained by GC-MS analysis, their retention time, % area, molecular formula, and molecular weight are listed in Table 4. The results of GC-MS evaluation showed the presence of many compounds including straight chain alkanes, fatty acids, aromatic compounds, and siloxanes.

**TABLE 4:** GC-MS analysis of methanol extract of *Brassica rapa*.

Sr no.	Compound name	Molecular formula	Molecular weight g/mol	% area	Retention time min
1	Hexadecanoic acid, methyl ester	C17H34O2	270.45	1.10	6.184
2	Methyl stearate	C19H38O2	298.50	0.91	7.199
3	7,10,13, hexadecatrienoic acid, methyl ester	C17H28O2	264.40	1.18	7.07
4	Hexadecane, 1-chloro	C16H33Cl	260.88	0.11	8.419
5	Methyl tetradecanoate	C15H30O2	242.39	0.08	5.084
6	Hexacosane	C26H54	366.70	0.06	9.253
7	Oleic acid	C18H34O2	282.46	0.09	6.546
8	Octacosane	C28H58	394.76	0.04	10.027
9	Cholesterol	C27H46O	386.65	0.10	11.295
10	Phenol, 4-ethenyl-2,6-dimethoxy	C10H12O3	180.20	0.21	4.317
11	Octadecane	C18H38	254.49	0.03	5.483
12	Disulfide, di-tert-dodecyl	C24H50 S2	402.8	0.06	4.909
13	1,2-benzene dicarboxylic acid ethyl methyl ester	C11H12O4	208.21	0.05	6.740
14	Pentacosane	C25H52	352.68	0.12	8.848
15	Dodecane, 2,6,10-trimethyl	C15H32	212.41	0.03	7.984
16	Androst-5-ene-3,4,17,triol	C28H54O3Si3	523	0.99	10.957
17	Isopropyl myristate	C17H34O2	270.45	0.10	5.628
18	Heptasiloxane	O6Si7	292.59	0.14	13.362
19	Cyclononasiloxane, octadecamethyl	C18H54O9Si9	667.38	1.88	10.105

#### 3.3.6 *In silico* molecular docking studies

**FIGURE 4:**
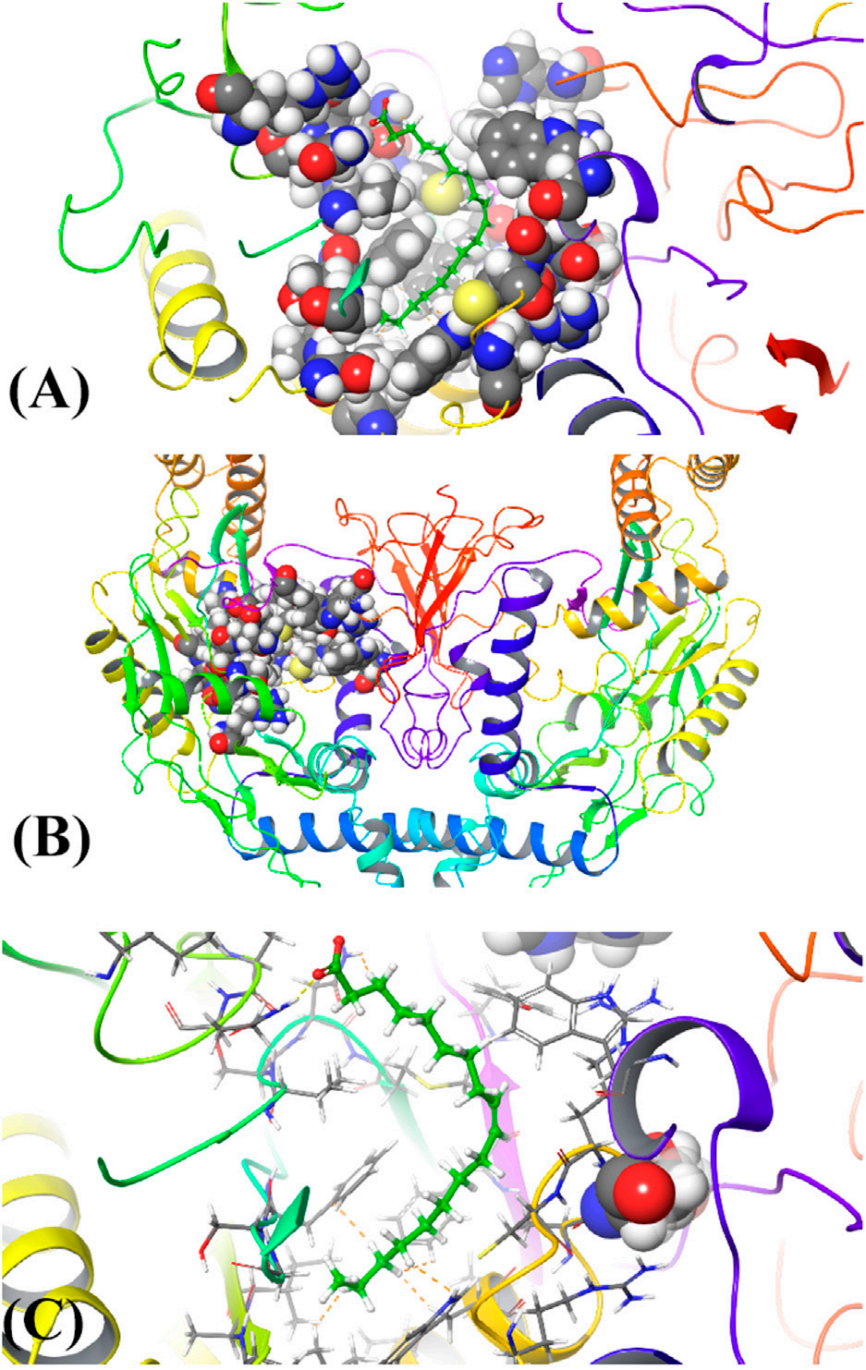
**(A)** 3D structure of ligand binding with c1 pocket of enzyme, **(B)** Horn shaped structure of enzyme with binding pocket, and **(C)** attachment of the ligand with the binding site of the enzyme.

**FIGURE 5:**
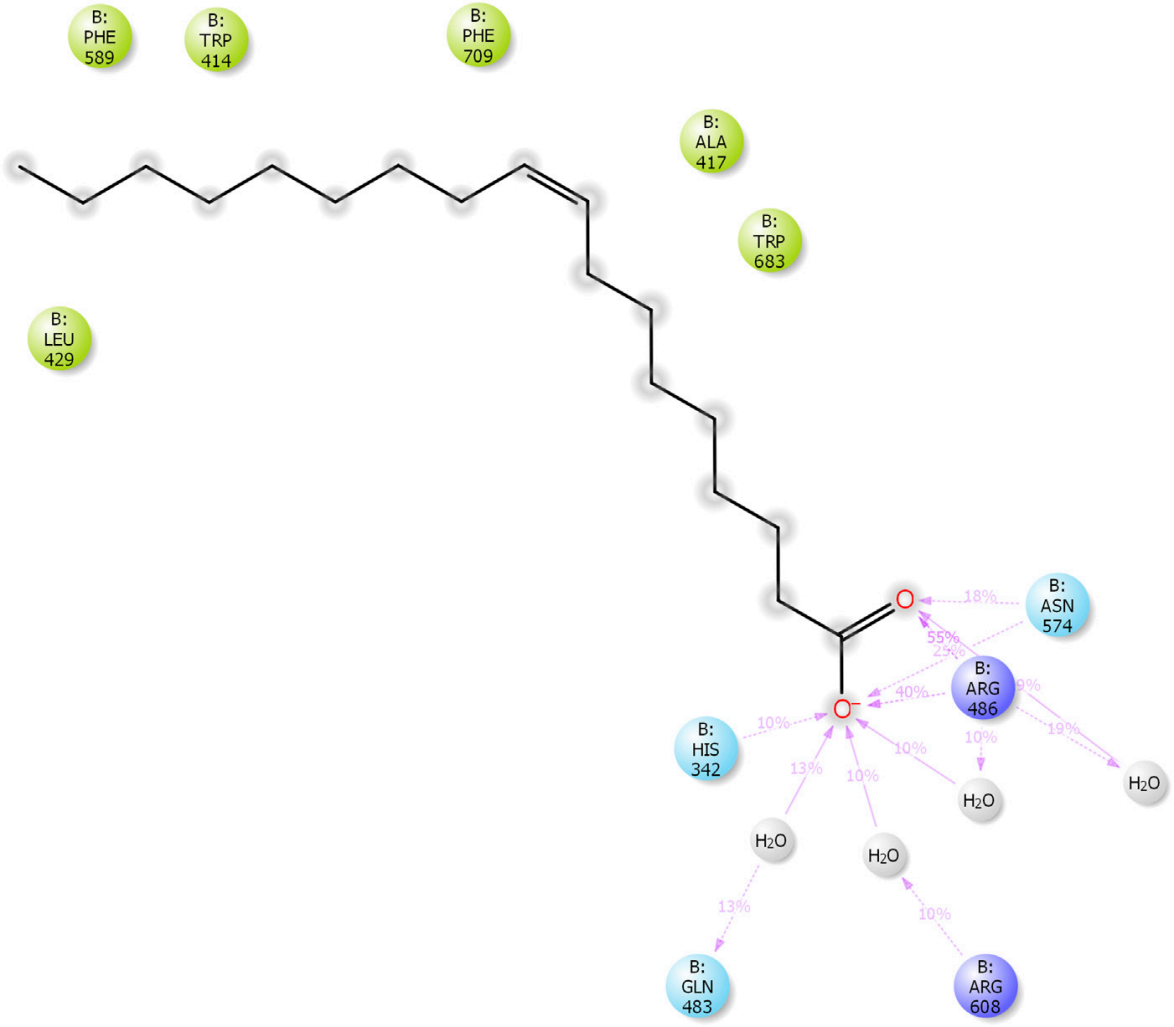
Two-dimensional ligand (oleic acid) interaction with endothelial nitric oxide synthase (eNOS). The binding of oleic acid takes place through amino acid residues; B:ARG 608, B:GLN483, B:HIS 342, and B:ARG 486.

**FIGURE 6:**
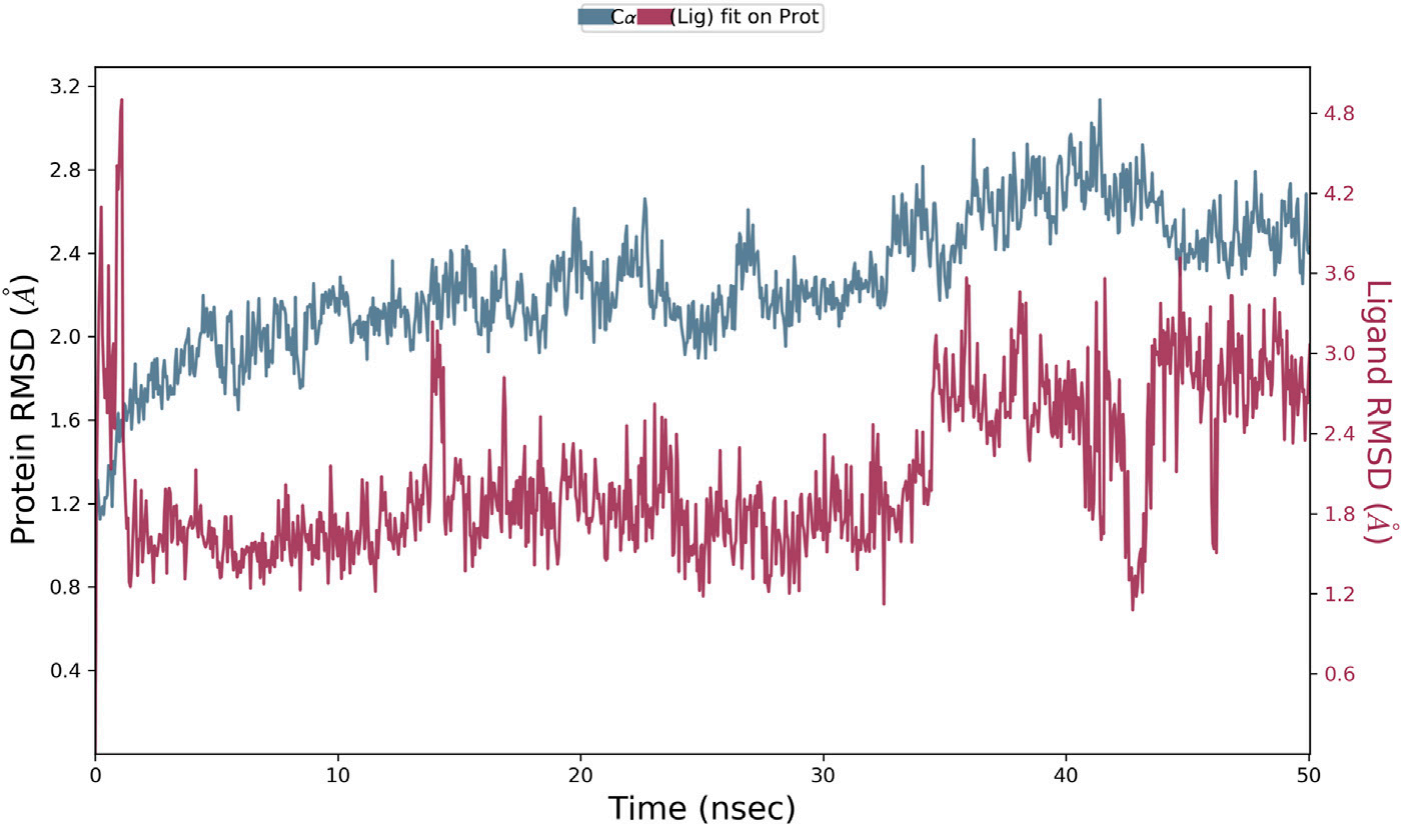
Root mean square deviation (RMSD) plot of protein and ligand.

**FIGURE 7:**
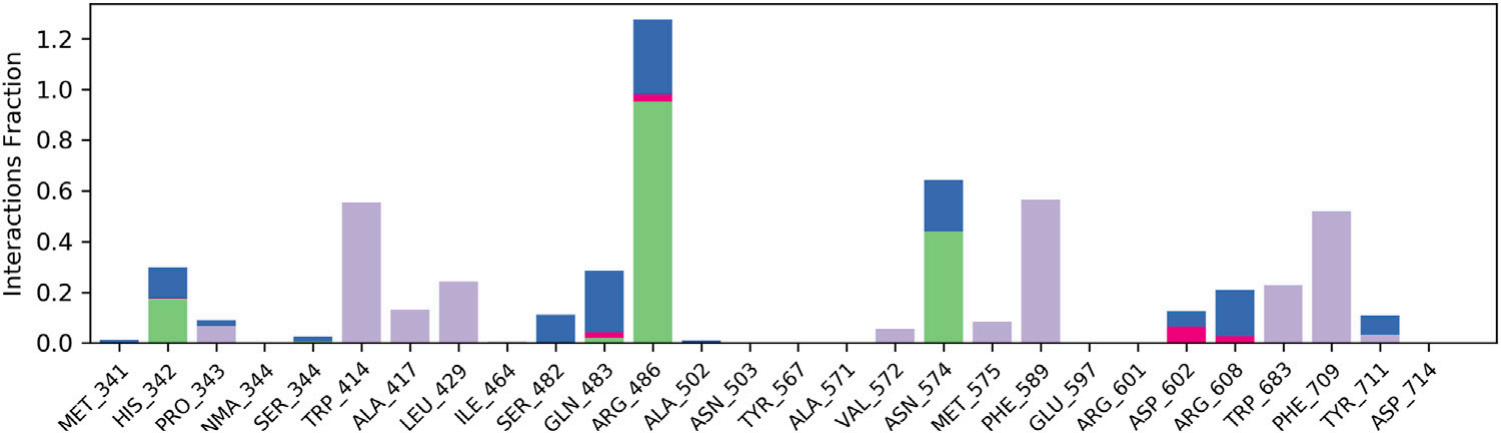
Plot of interaction fraction and amino acid residue interacting with the ligand. ARG 486 and ASN 574 bind with enzyme through strong hydrogen bonds. TRP 414, PHE 589, and PHE 709 showed hydrophobic interaction of simulation time between 50–60%. A water bridge mediated by hydrogen bonding is seen with ARG 486 residue.

The results of *in silico* docking studies are tabulated in Table 5. The escalated negative docking score (-4.818) depicts the high binding affinity (−51.3 kcal/mol) of the receptor–ligand complex portraying the biological activity of the compound. Figures 4A and B illustrate the 3D imaging of binding sites in the protein, while Figure 4C represents the binding site with ligand. The docking study reveals that oleic acid can bind within the C1 pocket of endothelial nitric oxide synthase depending upon the binding affinity toward that site shown in Figure 5. In eNOS, there are two binding pockets C1 and C2, where oleic acid can possibly bind through amino acid residues such as B:ARG 608, B:GLN483, B:HIS 342, and B:ARG 486. For testing the stability of the docked compound, docking simulations were performed with the help of the Maestro-Desmond v12.3 Schrödinger software package, and the interaction of eNOS with the appropriate ligand was evaluated. The addition of water molecules was carried out by placing the docking complex in an orthorhombic box. Na^+^/Cl^−^ ions were used to neutralize the charge. At normal pressure and 300 K temperature, the run time for docking simulation was 50 ns. Using the default setting, root mean square deviation (RMSD) of the protein–ligand complex and amino acids that participated in this contact were investigated. The RMSD plot of protein and ligand is shown in Figure 6. The binding of enzyme with the ligand against the interaction fraction displayed hydrogen bonding, hydrophobic and ionic interaction, and water bridges. ARG 486 and ASN 574 bind with the enzyme through strong hydrogen bonds. TRP 414, PHE 589, and PHE 709 showed hydrophobic interaction of simulation time between 50–60%. Water bridge-mediated hydrogen bonding is seen with ARG 486 residue. The binding residues in the ligand that react with the enzyme can be noted in Figure 7.

**TABLE 5:** Docking results showing G score, binding, and receptor energy.

Title	Docking score	Glide Gscore	Prime energy kcal/mol	Receptor energy kcal/mol	MMGBSA dg bind kcal/mol
Ligprep					
Oleic acid					
Glide dock					
1D0C					
Oleic acid	−4.818	−4.822			
Prime MMGBSA					
1D0C					
Oleic acid	−4.818	−4.822	−35774.8	−35687.247	−51.3
Desmond setup					
Oleic acid	−4.818	−4.822	−35774.8	−35687.247	−51.3

## 4 Discussion

The study was conducted on turnip leaves. Moisture content found in the leaves of *Brassica rapa* was 9.1% which was approximately similar to the findings of another study conducted on turnip leaves. Hammad and Abo-Zaid (2020) conducted a study in Egypt and observed the moisture content present in powdered turnip leaves to be 9.6%. An increase in moisture content increases microbial growth and decreases the storage time and efficacy of the product (Kunle et al., 2012). Less moisture content leads to increased stability (Oluyemisi et al., 2012). Turnip root powder has a percentage moisture content of 6.48 ± 0.45% as per a previous study (Mahfouz et al., 2019). Ash value is a measure of impurities present in the raw material used for drugs. The residual material that remains after the plant is incinerated gives the total ash value, while the presence of sand or silica in the plant gives the value of acid insolubility (Unit and Organization, 1992). The total percentage of ash in our sample, leaves of *Brassica rapa,* was 18% which is comparable to the results of a previous study which revealed that the ash value of powdered turnip leaves in Egypt was 12.12% (Hammad and Abo-Zaid, 2020).

Primary metabolites (carbohydrates, proteins, and lipids) are involved in plant maturation and growth. (Erb and Kliebenstein, 2020). Amongst other factors, the development and growth of an individual depends on the number of carbohydrates, proteins, and lipids present in the diet. Proteins are important in performing different body functions, for instance, nutrients transported across the cell membrane and the functioning of enzymes (Mæhre et al., 2018). According to Hammad and Abo-Zaid (2020), the percentage of fats, carbohydrates, and proteins detected in powdered leaves of *Brassica rapa* was1.69, 41.92, and 16.44%, respectively. Our calculated values of lipid, carbohydrates, and protein are 3.48, 57.45, and 11.90%, respectively. This shows that turnip leaves have high carbohydrate content. Carbohydrates are remarkably essential in plant cell morphology as well as in metabolic processes. They mostly occur as polysaccharides and serve as anti-ulcer, laxative, hypoglycemic, immunomodulators, analgesic, expectorant, hypocholesterolemic, and anabolic. They are advantageous in decreasing the toxicity of cytostatic drugs and antibiotics. The most important function is their role in the pharmacodynamics of drugs obtained from plant source (Budniak et al., 2021).

Because of their scavenging power, polyphenols are considered essential constituents of plants (Nguyen et al., 2020). Other than their role as antioxidants, flavonoids are also responsible for several therapeutic properties such as antitumor, anti-inflammatory, antiviral, antibacterial, and anti-allergy (Priecina and Karlina, 2013). Our research suggests that the methanolic extract of turnip was rich in flavonoids and polyphenols.

Glycosaponins are secondary metabolites with a large molecular weight. They are of prime importance for their cardio-protective and anti-angiogenesis inhibition of multi-drug resistance effects. Saponins play a vital role in minimizing the effects of radio and chemotherapy (Xu et al., 2016). This study indicates that aqueous extracts of *Brassica rapa* contained glycosaponins in abundance, while glycosaponins were not detected in non-polar solvents (n-hexane and chloroform).

As stated by Chizoruo et al. (2019), the functional groups in the IR region indicate the existence of phytoconstituents in plant studies. The presence of alcohols, aldehydes, ketones, carboxylic acids, aromatic aldehydes, ethers, nitro compounds, alkanes, alkenes, and amines was shown by FTIR analysis on turnip leaves. A similar study was performed by Dhivya (2017), and our results are also in accordance with the reports of Chizoro et al.

The results of GC-MS evaluation showed the presence of many compounds, most of which are of great importance in the field of medicine. The constituents include straight chain alkanes such as pentacosane, hexacosane, octacosane, octadecane, and hexadecane. Pentacosane exhibits medicinal properties by acting as a 5-alpha reductase inhibitor, TNF alpha-inhibitor, and increasing the activity of alpha-mannosidase (Gomathi Priyadarshini et al., 2017), whereas hexacosane has been reported to have antifungal activity (Adeyemi et al., 2017). Studies on octacosane revealed its mosquitocidal effect (Rajkumar and Jebanesan, 2004) and is a compound with antimicrobial properties (Rai et al., 2021). Dodecane 2,6,10-trimethyl is an alkane that is antibacterial in nature (Nahid et al., 2012). The presence of cholesterol, reported in our study, is known for its antimicrobial activity (Sribalan et al., 2016). Hexadecanoic acid, methyl ester is employed as an antioxidant, 5-alpha reductase inhibitor, and hypocholesterolemic (Priya and Subhashini, 2016). Methyl tetradecanoate is an ester of myristic acid that possesses antioxidant, anticancer, nematicidal, and hypocholesterolemic properties (Elaiyaraja and Chandramohan, 2016). Isopropyl myristate is a long-chain fatty ester famous for its use in topical preparation and as a moisturizer (Chandrasekar et al., 2015). Methyl stearate, an ester of fatty acid, shows antifungal and antioxidant properties (Javaid et al., 2021). Siloxanes such as octadecamethyl, cyclononasiloxane, and heptasiloxane have applications in implants and skin patches (Neha et al., 2021). Research revealed that oleic acid has antimicrobial and antifungal properties (Naqvi et al., 2020). It is also used in the preparation of anticancer and antihypertensive medicines (Funari et al., 2003).

GC-MS analysis of methanol extract of *Brassica rapa* reported the presence of oleic acid as the bioactive compound. As per research conducted by Zhang et al. (2010), oleic acid is responsible for the lowering of blood pressure induced by angiotensin II *via* ANG II signal modulation in the blood vessels. In an *in vivo* study, a substantial reduction occurred in the number of inhibitory G-proteins when the rats were treated with oleic acid, causing an increase in vasodilation (Teres et al., 2008). Our study has also indicated that oleic acid could inhibit ACE.

In conclusion, we applied the *in silico* molecular modeling methods to verify our results whereby oleic acid was subjected to molecular docking. Excellent binding of oleic acid was observed with the enzyme endothelial nitric oxide synthase (eNOS) which showed its antihypertensive potential. Our research is based on computational data that is in coherence with the experimental results. Some other compounds have also been detected in GC-MS, which can be further evaluated and docked for the purpose of designing a potent antihypertensive drug.

## Data Availability

The raw data supporting the conclusion of this article will be made available by the authors, without undue reservation.

## References

[B1] Adeyemi M. Ekunseitan D. Abiola S. Dipeolu M. Egbeyale L. Sogunle O. (2017). Phytochemical analysis and GC-MS determination of Lagenaria breviflora R. fruit. Int. J. Pharmacogn. Phytochem. Res. 9, 1045–1050. 10.25258/phyto.v9i07.11178

[B2] Ahmad K. Jafar T. H. (2005). Prevalence and determinants of blood pressure screening in Pakistan. J. Hypertens. 23, 1979–1984. 10.1097/01.hjh.0000187258.86824.00 16208138

[B3] Ahmadvand S. Sariri R. (2008). Antimicrobial activity of crude extracts of turnip (Brassica rapa). J. Pure Appl. Microbiol. 2, 193–196.

[B4] Al-Hooti S. Sidhu J. Qabazard H. (1997). Physicochemical characteristics of five date fruit cultivars grown in the United Arab Emirates. Plant Foods Hum. Nutr. 50, 101–113. 10.1007/BF02436030 9201745

[B5] Beltagy A. M. (2014). Investigation of new antimicrobial and antioxidant activities of Brassica rapa L. Int. J. Pharm. Pharm. Sci. 6, 84–88.

[B6] Besbes S. Blecker C. Deroanne C. Drira N.-E. Attia H. (2004). Date seeds: Chemical composition and characteristic profiles of the lipid fraction. Food Chem. 84, 577–584. 10.1016/s0308-8146(03)00281-4

[B7] Budniak L. Slobodianiuk L. Marchyshyn S. Ilashchuk P. (2021). Determination of polysaccharides in Gentiana cruciata L. herb. Pharmacologyonline 2, 1473–1479.

[B8] Burt V. L. Whelton P. Roccella E. J. Brown C. Cutler J. A. Higgins M. (1995). Prevalence of hypertension in the US adult population: Results from the third national health and nutrition examination survey, 1988-1991. Hypertension 25 (3), 305–313. 10.1161/01.hyp.25.3.305 7875754

[B9] Carretero O. A. Oparil S. (2000). Essential hypertension: Part I: Definition and etiology. Circulation 101, 329–335. 10.1161/01.cir.101.3.329 10645931

[B10] Chandrasekar T. Rao M. R. K. Kumar R. V. Prabhu K. Kumar S. N. Divya D. (2015). GC-MS analysis, antimicrobial, antioxidant activity of an Ayurvedic medicine, Nimbapatradi choornam. J. Chem. Pharm. Res. 7, 124–136.

[B11] Chang C.-C. Yang M.-H. Wen H.-M. Chern J.-C. (2002). Estimation of total flavonoid content in propolis by two complementary colorimetric methods. J. food drug analysis 10.

[B12] Chizoruo I. F. Onyekachi I. B. Ebere E. C. (2019). Trace metal, FTIR and phytochemical analysis of Viscum album leaves harvested from Pentaclethra macrophylla. Poland: World News of Natural Sciences, 25.

[B13] Comini L. Bachetti T. Cargnoni A. Bastianon D. Gitti G. L. Ceconi C. (2007). Therapeutic modulation of the nitric oxide: All ace inhibitors are not equivalent. Pharmacol. Res. 56 (1), 42–48. 10.1016/j.phrs.2007.03.004 17475504

[B14] Cushman D. W. Cheung H. S. (1971). Spectrophotometric assay and properties of the angiotensin-converting enzyme of rabbit lung. Biochem. Pharmacol. 20, 1637–1648. 10.1016/0006-2952(71)90292-9 4355305

[B15] Dhivya K. (2017). Screening of phytoconstituents, UV-VIS spectrum and FTIR analysis of micrococca mercurialis (L.) benth. Int. J. Herb. Med. 5, 40–44.

[B16] Elaiyaraja A. Chandramohan G. (2016). Comparative phytochemical profile of Indoneesiella echioides (L.) Nees leaves using GC-MS. J. Pharmacogn. Phytochemistry 5, 158.

[B17] Erb M. Kliebenstein D. J. (2020). Plant secondary metabolites as defenses, regulators, and primary metabolites: The blurred functional trichotomy. Plant Physiol. 184 (1), 39–52. 10.1104/pp.20.00433 32636341 PMC7479915

[B18] Funari S. S. Barceló F. Escribá P. V. (2003). Effects of oleic acid and its congeners, elaidic and stearic acids, on the structural properties of phosphatidylethanolamine membranes. J. Lipid Res. 44, 567–575. 10.1194/jlr.M200356-JLR200 12562874

[B19] Gomathi Priyadarshini A. A. E. Anthony J. Rao M. R. K. Prabhu K. Ramesh A. Krishna V. (2017). The GC MS analysis of one medicinal plant, Premna tomentosa. J. Pharm. Sci. Res. 9, 1595–1597.

[B20] Hammad E. M. Abo-Zaid E. M. (2020). Properties of noodles fortification with turnip leave powder. J. Food Dairy Sci. 11, 209–213. 10.21608/jfds.2020.111755

[B21] Hussain K. Ismail Z. Sadikun A. Ibrahim P. (2008). Analysis of proteins, polysaccharides, glycosaponins contents of Piper sarmentosum Roxb. and anti-TB evaluation for bio-enhancing/interaction effects of leaf extracts with Isoniazid (INH).

[B22] Javaid A. Khan I. H. Ferdosi M. F. (2021). Antimicrobial and other bioactive constituents of cannabis sativus roots from Pakistan. Pak. J. Weed Sci. Res. 27, 359–368. 10.28941/pjwsr.v27i3.984

[B23] Kunle O. F. Egharevba H. O. Ahmadu P. O. (2012). Standardization of herbal medicines-A review. Int. J. Biodivers. Conserv. 4 (3), 101–112. 10.5897/ijbc11.163

[B24] Lifton R. P. Gharavi A. G. Geller D. S. (2001). Molecular mechanisms of human hypertension. Cell 104, 545–556. 10.1016/s0092-8674(01)00241-0 11239411

[B25] Lowry O. H. Rosebrough N. J. Farr A. L. Randall R. J. (1951). Protein measurement with the Folin phenol reagent. J. Biol. Chem. 193, 265–275. 10.1016/s0021-9258(19)52451-6 14907713

[B26] Mahfouz M. Abd-Elnoor A. V. EL-Razek A. Ragwa I. (2019). The influence of adding turnip roots (Brassica rapa var. rapa L.) powder on the antioxidant activity and acrylamide content in some fried foods. Alexandria Sci. Exch. J. 40, 717–730. 10.21608/asejaiqjsae.2019.68841

[B27] Mæhre H. K. Dalheim L. Edvinsen G. K. Elvevoll E. O. Jensen I.-J. (2018). Protein determination—Method matters. Foods 7, 5. 10.3390/foods7010005 PMC5789268 29301260

[B28] Messerli F. H. Williams B. Ritz E. (2007). Essential hypertension. Lancet 370, 591–603. 10.1016/S0140-6736(07)61299-9 17707755

[B29] Mittal B. V. Singh A. K. (2010). Hypertension in the developing world: Challenges and opportunities. Am. J. Kidney Dis. 55, 590–598. 10.1053/j.ajkd.2009.06.044 19962803

[B30] Nahid R. Ali S. Farshid S. (2012). Antimicrobial activity and constituents of the hexane extracts from leaf and stem of Origanum vulgare L. ssp. Viride (Boiss.) Hayek. growing wild in Northwest Iran. J. Med. Plants Res. 6, 2681–2685.

[B31] Naqvi S. F. Khan I. H. Javaid A. (2020). Hexane soluble bioactive components of Chenopodium murale stem. Pak. J. Weed Sci. Res. 26, 425–432. 10.28941/pjwsr.v26i4.875

[B32] Neha B. Jannavi R. Sukumaran P. (2021). Phyto-pharmacological and biological aspects of vitex negundo medicinal plant-A review. Cardiovasc. Dis. 6, 7.

[B33] Nguyen N. Nguyen M. Nguyen V. LE V. Trieu L. LE X. (2020). “The effects of different extraction conditions on the polyphenol, flavonoids components and antioxidant activity of Polyscias fruticosa roots,” in IOP conference series: Materials science and engineering (Bristol: IOP Publishing), 022067.

[B34] Norlander A. E. Madhur M. S. Harrison D. G. (2018). The immunology of hypertension. J. Exp. Med. 215, 21–33. 10.1084/jem.20171773 29247045 PMC5748862

[B35] Oluyemisi F. Henry O. Peter O. (2012). Standardization of herbal medicines-A review. Int. J. Biodivers. Conservation 4, 101–112.

[B36] Paul S. Geng C. A. Yang T. H. Yang Y. P. Chen J. J. (2019). Phytochemical and health‐beneficial progress of turnip (Brassica rapa). J. Food Sci. 84, 19–30. 10.1111/1750-3841.14417 30561035

[B37] Priecina L. Karlina D. (2013). “Total polyphenol, flavonoid content and antiradical activity of celery, dill, parsley, onion and garlic dried in conventive and microwave-vacuum dryers,” in 2nd international conference on nutrition and food Sciences, 107–112.

[B38] Priya S. Subhashini A. (2016). Phytochemical screening and GCMS analysis of methanolic extract of leaves of Pisonia aculeata Linn. Int. J. Pharma Bio Sci. 7, 317–322. 10.22376/ijpbs.2016.7.4.p317-322

[B39] Rai R. Singh R. K. Suthar S. (2021). Production of compost with biopesticide property from toxic weed lantana: Quantification of alkaloids in compost and bacterial pathogen suppression. J. Hazard. Mat. 401, 123332. 10.1016/j.jhazmat.2020.123332 32763675

[B40] Rajkumar S. Jebanesan A. (2004). Mosquitocidal activities of octacosane from moschosma polystachyum linn.(lamiaceae). J. Ethnopharmacol. 90, 87–89. 10.1016/j.jep.2003.09.030 14698514

[B41] Sheng-Ji P. (2001). Ethnobotanical approaches of traditional medicine studies: Some experiences from asia. Pharm. Biol. 39 (1), 74–79. 10.1076/phbi.39.s1.74.0005 21554174

[B42] Shoeb M. (2006). Anti-cancer agents from medicinal plants. Bangladesh J. Pharmacol. 1, 35–41. 10.3329/bjp.v1i2.486

[B43] Siddiqui M. Ismail Z. Sahib H. Helal M. Abdul M. A. (2009). Analysis of total proteins, polysaccharides and glycosaponins contents of orthosiphon stamineus benth. In spray and freeze dried methanol: Water (1: 1) extract and its contribution to cytotoxic and antiangiogenic activities. Pharmacogn. Res. 1.

[B44] Slinkard K. Singleton V. L. (1977). Total phenol analysis: Automation and comparison with manual methods. Am. J. enology Vitic. 28, 49–55.

[B45] Sribalan R. Padmini V. Lavanya A. Ponnuvel K. (2016). Evaluation of antimicrobial activity of glycinate and carbonate derivatives of cholesterol: Synthesis and characterization. Saudi Pharm. J. 24, 658–668. 10.1016/j.jsps.2015.05.003 27829808 PMC5094438

[B46] Taveira M. Fernandes F. DE Pinho P. G. Andrade P. B. Pereira J. A. Valentão P. (2009). Evolution of Brassica rapa var. rapa L. volatile composition by HS-SPME and GC/IT-MS. Microchem. J. 93, 140–146. 10.1016/j.microc.2009.05.011

[B47] Teres S. Barceló-Coblijn G. Benet M. Alvarez R. Bressani R. Halver J. E. (2008). Oleic acid content is responsible for the reduction in blood pressure induced by olive oil. Proc. Natl. Acad. Sci. U. S. A. 105 (37), 13811–13816. 10.1073/pnas.0807500105 18772370 PMC2544536

[B48] Unit P. Organization W. H. (1992). Quality control methods for medicinal plant materials. France: World Health Organization.

[B49] USP (2005). United States pharmacopoeia D-1. Washington: Drug Information for the health care professional, 1. Thomas PDR. Micromedex.

[B50] Wahedi H. M. Ahmad S. Abbasi S. W. (2021). Stilbene-based natural compounds as promising drug candidates against COVID-19. J. Biomol. Struct. Dyn. 39, 3225–3234. 10.1080/07391102.2020.1762743 32345140

[B51] Whelton P. K. (1994). Epidemiology of hypertension. Lancet (London, Engl. 344 (8915), 101–106. 10.1016/s0140-6736(94)91285-8 7912348

[B52] Williams B. (2009). The aorta and resistant hypertension. Washington, DC: American College of Cardiology Foundation. 10.1016/j.jacc.2008.10.027 19179204

[B53] Xu X. H. Li T. Fong C. M. V. Chen X. Chen X. J. Wang Y. T. (2016). Saponins from Chinese medicines as anticancer agents. Molecules 21 (10), 1326. 10.3390/molecules21101326 PMC6272920 27782048

[B54] Yadav R. Agarwala M. (2011). Phytochemical analysis of some medicinal plants. J. phytology 3, 1.

[B55] Zhang J. Villacorta L. Chang L. Fan Z. Hamblin M. Zhu T. (2010). Nitro-oleic acid inhibits angiotensin II–induced hypertension. Circ. Res. 107 (4), 540–548. 10.1161/CIRCRESAHA.110.218404 20558825 PMC2937264

